# Predicted dominance of variant Delta of SARS-CoV-2 before Tokyo Olympic Games, Japan, July 2021

**DOI:** 10.2807/1560-7917.ES.2021.26.27.2100570

**Published:** 2021-07-08

**Authors:** Kimihito Ito, Chayada Piantham, Hiroshi Nishiura

**Affiliations:** 1 International Institute for Zoonosis Control, Hokkaido University, Hokkaido, Japan; 2 Graduate School of Infectious Diseases, Hokkaido University, Hokkaido, Japan; 3 Graduate School of Medicine, Kyoto University, Kyoto, Japan

**Keywords:** SARS-CoV-2, Tokyo Olympic Games, Delta variant, relative instantaneous reproduction numbers, R_RI_

## Abstract

Using numbers of SARS-CoV-2 variants detected in Japan as at 13 June 2021, relative instantaneous reproduction numbers (R_RI_) of the R.1, Alpha, and Delta variants with respect to other strains circulating in Japan were estimated at 1.25, 1.44, and 1.95. Depending on the assumed serial interval distributions, R_RI_ varies from 1.20–1.32 for R.1, 1.34–1.58 for Alpha, and 1.70–2.30 for Delta. The frequency of Delta is expected to take over Alpha in Japan before 23 July 2021.

Severe acute respiratory syndrome coronavirus 2 (SARS-CoV-2), the causative agent of coronavirus disease (COVID-19), has undergone adaptive evolution since its emergence in the human population in 2019. On 31 May 2021, the World Health Organization (WHO) has designated four variants of SARS-CoV-2 as variants of concern (VOC)— Alpha, Beta, Gamma, and Delta corresponding to the Phylogenetic Assignment of Named Global Outbreak (Pango) lineage designation B.1.1.7, B.1.351, P.1 and B.1.617.2, respectively [1]. 

Multiple SARS-CoV-2 variants are circulating in Japan and because of the high transmissibility of the VOC, the replacement of locally circulating strains by Alpha and Delta VOC poses a serious public health threat in Japan. Here we used a renewal-equation-based model to describe the adaptive evolution among multiple variants, i.e., R.1, Alpha and Delta variants in addition to ordinary variant, in the country to inform risk-assessment ahead of the Summer Olympic Games in Tokyo starting on 23 July 2021.

## Epidemiological situation in Japan in June/July 2021

In Japan, in the middle of March 2021, COVID-19 case numbers increased and a fourth wave began when the SARS-CoV-2 Alpha VOC and the lineage R.1 appeared. R.1 (Pangolin designation) is a local mutant possessing an E484K mutation on its spike protein [2]. The Japanese government declared a state of emergency on 25 April in 10 of the 47 prefectures in Japan. During the fourth wave in Japan, public health and social measures against COVID-19 included the closure of restaurants, cancellation of mass gathering events and requests to ‘stay home’. New cases decreased in early May and the emergency state in Tokyo was lifted on 20 June, but new cases in Tokyo started increasing again hereafter [3]. As at 20 June, the R.1 variant, Alpha and Delta VOC are circulating in Japan in addition. 

## Modelling advantageous selection among multiple SARS-CoV-2 variants

Using the numbers of SARS-CoV-2 variants found in the GISAID database [4] and those detected by PCR in Tokyo, we estimate the relative instantaneous reproduction numbers (*R_RI_*) of the R.1 variant and the Alpha and Delta VOC with respect to other strains circulating in Japan before the introduction of the Alpha and Delta VOC and R.1. We also show the expected temporal changes in variant relative frequencies of SARS-CoV-2 in Japan until early August 2021.

Suppose that we have a large population of viruses consisting of lineages *a*, *A*_1_, …, *A_n_*, of which frequencies in the viral population at a calendar time *t* are *q_a_*(*t*), *q_A_*_1_(*t*), …, *q_An_*(*t*), respectively. Suppose also that *a* is a baseline lineage that was circulating at the beginning of the target period of analysis and that *A*_1_, …, *A_n_* are lineages introduced to the population at times *t*_1_, …, *t_n_*, respectively.

We assume that viruses of lineages *A*_1_, …, *A_n_* generate 1+*s*_1_, …, 1+*s_n_* times as many secondary transmissions as those of the baseline lineage *a*, respectively. The instantaneous reproduction number is defined as the average number of people an infected individual at time *t* could be expected to infect given that conditions remain unchanged [5]. Let *I*(*t*) be the total number of new infections by viruses of any lineages of *a* or *A*_1_, …, *A_n_* at calendar time *t* and *g*(*j*) be the probability mass function of the serial interval. Suppose that *g*(*j*) is small enough to be neglected for *j*<1 or *j*>*l*. The instantaneous reproduction numbers of lineage *a* and *A*_1_, …, *A_n_* at calendar time *t* are represented as follows:

$$R_{a}\left(t\right)=\;\frac{q_{a}\left(t\right)I(t)}{\sum_{j=1}^{l}g\left(j\right)q_{a}\left(t-j\right)I(t-j)}\;\;\;\;\;\;\;\;(1)\;$$

$$R_{A_{i}}(t)=\;\frac{q_{A_{i}}(t)I(t)}{\sum_{j=1}^{l}g\left(j\right)q_{A_{i}}\left(t-j\right)I(t-j)}\;\;\;\;\;\;\;\;(2)$$

Since a virus of lineage *A_i_* generates 1+*s_i_* times as many secondary transmissions as those of lineage *a*, the following equation holds

$$R_{A_{i}}\left(t\right)=\left(1+s_{i}\right)R_{a}\left(t\right)\;\;\;\;\;\;\;\;(3)$$

for each calendar time *t* ≥ *t_i_*. We call the value of 1+*s_i_* the *R_RI_* of *A_i_* with respect to *a*.

To allow an explicit statistical estimation of the *R_RI_*, here we impose an assumption, i.e. within a single generation of transmission from calendar time t–*l* to t–1, the incidence (i.e. the number of new infections) did not greatly vary and they can be approximated to be equal such as

$$I\left(t-1\right)≃I\left(t-2\right)≃\cdots ≃I\left(t-l\right)\;\;\;\;\;\;\;(4)$$

The approximation in Formula (4) does not necessarily mean that the reproduction number was a constant over calendar time. Rather, the assumption of ‘close incidence’ allows us to take *I*(*t*) from the summation part of the renewal equation and allows us to cancel it out. The frequency of lineage *A_i_* in the viral population at calendar time *t*, *q_Ai_*(*t*), is now modelled as

$$q_{A_{i}}\left(t\right)=\;\frac{q_{A_{i}}\left(t\right)I(t)}{q_{a}\left(t\right)I\left(t\right)+\sum_{i=1}^{n}q_{A_{i}}\left(t\right)I(t)}$$

$$=\;\frac{\sum_{j=1}^{l}{g(j)R}_{A_{i}}\left(t\right)q_{A_{i}}(t-j)I(t-j)}{\sum_{j=1}^{l}g\left(j\right)R_{a}(t)q_{a}(t-j)I(t-j)+\sum_{i=1}^{n}\sum_{j=1}^{l}g\left(j\right)R_{A_{i}}\left(t\right)q_{A_{i}}(t-j)I(t-j)}$$

$$=\;\frac{\sum_{j=1}^{l}g(j)\left(1+s_{i}\right)q_{A_{i}}(t-j)}{1+\sum_{i=1}^{n}\sum_{j=1}^{l}g\left(j\right)s_{i}q_{A_{i}}(t-j)}\;\;\;\;\;\;\;\;(5)$$

## Numbers of SARS-CoV-2 variants detected in Japan

We downloaded metadata of sequences of SARS-CoV-2 submitted from Japan since 1 December 2020 from the GISAID EpiCoV database [4] on 16 June 2021. The sequencing rate in Japan in December 2020 was 0.06 sequences per case (4,826 sequences / 83,544 cases). After removing sequence records of viruses detected at the airport quarantine stations in Japan, Pango lineage labels [2] assigned to these sequences were collected (Supplementary Table 1). Numbers of sequences assigned to the R.1 variant as well as to Alpha (B.1.1.7) or Delta (B.1.617.2) VOC were counted and those of other lineages were summed up to ‘other’ lineages. Weekly numbers of the R.1 and the Alpha and Delta VOC detected by PCR in Tokyo were obtained from the report on COVID-19 monitoring submitted by the Tokyo Metropolitan Government on 17 June 2021 [6]. 

The PCR detection counts until 25 April and the GISAID variant counts from 26 April 2021 were excluded from the analysis to avoid double counting. Finally, daily variant frequencies of a total of 28,211 sequences—consisting of 5,186 R.1, 8,205 Alpha, 2 Delta, and 14,818 other SARS-CoV-2 viruses—and weekly variant frequencies of 784 viruses—consisting of 88 R.1, 664 Alpha, and 32 Delta variants were obtained and used in the rest of analyses (Supplementary Table 2; Supplementary Table 3).

### Relative instantaneous reproduction numbers

Let *N*(*t*) be the total number of sequences of lineages *A*_1_, …, *A_n_* or *a* observed at calendar time *t*, and let *d*_1_, …, *d_k_* be calendar times such that *N*(*d_j_*) > 0 for 1 ≤ *j* ≤ *k*. Suppose that we have *N_Ai_*(*d_j_*) sequences of lineage *A_i_* at calendar time *d_j_* for 1 ≤ *i* ≤ *n* and 1 ≤ *j* ≤ *k* . Since lineage *A_i_* emerged at time *t_i_*, *q_Ai_*(*d_j_*)=0 for *d_j_*<*t_i_*. If the lineage *A_i_* emerges with an initial frequency of *q_Ai_*(*t_i_*) at calendar time *t_i_*, then the likelihood function of parameters *s*_1_, …, *s_n_* and *q*_1_(*t*_1_), …, *q_n_*(*t_n_*) for observing *N_Ai_*(*d*_1_), …, *N_A_*_1_(*d_k_*), …, *N_An_*(*d*_1_), …, *N_An_*(*d_k_*) sequences of lineages *A*_1_, …, *A_n_*, at calendar times *d*_1_, …, *d_k_* is given by the following formula:

$$L\left(s_{1},\cdots ,s_{n},q_{1}(t_{1}\right),\cdots ,q_{n}\left(t_{n}\right);\;N_{A_{1}}\left(d_{1}\right),\cdots ,N_{A_{1}}\left(d_{k}\right),\cdots N_{A_{n}}\left(d_{1}\right),\cdots ,N_{A_{n}}\left(d_{k}\right)\;)$$

$$=\prod_{j=1}^{k}\frac{N\left(d_{j}\right)!}{N_{a}\left(d_{j}\right)!\prod_{i}^{n}N_{A_{i}}\left(d_{j}\right)!}q_{a}{\left(d_{j}\right)}^{N_{a}(d_{j})}\prod_{i=1}^{n}q_{A_{i}}{\left(d_{j}\right)}^{N_{A_{i}}(d_{j})}\;\;\;\;\;\;\;\;(6)$$

$$\text{where}\;N_{a}\left(d_{j}\right)=N\left(d_{j}\right)-\sum_{i=1}^{n}N_{A_{i}}\left(d_{j}\right)\text{ for }1\leq j\leq k\text{.}$$

The earliest dates of the R.1 variant and the Alpha and Delta VOC among the GISAID sequences from Japan (excluding those from the airport quarantine stations) were 1 December and 15 December 2020, and 15 April 2021, respectively. We assume that *t*_R.1_, *t*_Alpha_, and *t*_Delta_ are these dates. We used fixed dates for *t*_R.1_, *t*_Alpha_, and *t*_Delta_ because the estimation of the importation day of the Alpha or Delta VOC is not the purpose of this study. Undetected viruses before *t*_R.1_, *t*_Alpha_, and *t*_Delta_ do not affect the analysis largely because increased values of *q*_R.1_(*t*_R.1_), *q*_Alpha_(*t*_Alpha_) and *q*_Delta_(*t*_Delta_), can compensate for the effect of these undetected viruses. 

The serial intervals were assumed to follow a lognormal distribution with log mean (*μ*) = 1.38 and log standard deviation (*σ*) = 0.563, discretised and truncated so that *g*(0) = 0 and *g*(*j*) = 0 for j > 0. Parameters *s*_R.1_, *s*_Alpha_, *s*_Delta_, *q*_R.1_(*t*_R.1_), *q*_Alpha_(*t*_Alpha_), and *q*_Delta_(*t*_Delta_) were estimated by maximising the likelihood defined in Formula (6). The 95% confidence intervals (CIs) of these parameters were estimated by profile likelihood [7]. The optimisation of likelihood function and calculation of 95% CIs were performed using the nloptr package in R [8,9].

The selective advantages *s*_R.1_, *s*_Alpha_ and *s*_Delta_, with respect to other strains circulating in Japan were estimated to be 0.25 (95% CI: 0.25–0.25), 0.44 (95% CI: 0.43–0.44) and 0.95 (95% CI: 0.90–0.99) (Table 1). The initial frequencies, *q*_R.1_(*t*_R.1_), *q*_Alpha_(*t*_Alpha_), and *q*_Delta_(*t*_Delta_), were estimated as shown in Table 1. The *R_RI_* of the Delta VOC in Japan was compatible with the estimate of 1.97 (95%CI: 1.76–2.17), which was obtained using global data by Campbell et al. [10]. However, the *R_RI_* of the Alpha VOC in Japan was not within the 95%CI from the estimate of 1.29 (95%CI: 1.24–1.33) from the same study. The discrepancy of *R_RI_* among countries has been observed in the same study, and we suspect that it may be attributed to the difference in the transmissibility of the baseline viruses, with which *R_RI_* is calculated. 

**Table 1:** Estimated selective advantages and initial frequencies for SARS-CoV-2 R1 variant and Alpha and Delta VOC compared with other circulating variants, Japan, July 2021

Parameter	Estimated values	95% confidence interval
*^S^R.*1	0.25	0.25–0.25
*^S^Alpha*	0.44	0.44–0.44
*^S^Delta*	0.95	0.90–0.99
*^q^R.*1	0.0043	0.0041–0.0046
*^q^Alpha*	0.0011	0.0010–0.0012
*^q^Delta*	0.0029	0.0023–0.0033

s: selective advantage; q: initial relative frequencies.

**Figure 1:**
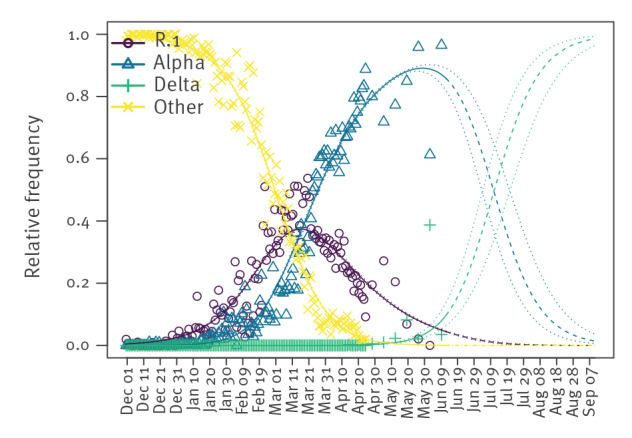
Estimated temporal changes in SARS-CoV-2 variant relative frequencies of R.1, Alpha and Delta VOC and other strains circulating, Japan, 1 December 2020 to 11 September 2021

CI: confidence interval; SARS-CoV-2: severe acute respiratory syndrome coronavirus 2; VOC: variants of concern.

Figure 1 shows temporal changes in frequencies of the R.1 variant, Alpha and Delta VOC and other lineages circulating in Japan estimated by our model. The Alpha VOC became dominant (frequency > 50%) in Japan at the end of May 2021. The Delta VOC is predicted to become dominant over Alpha on 12 July 2021 (95% CI: 5 July to 22 July).

The circles, triangles, pluses, and crosses respectively indicate frequencies of SARS-CoV-2 sequences of R.1 variant, Alpha and Delta VOC, and others collected in Japan from 1 December 2020. Daily frequencies are shown until 25 April 2021 and weekly frequencies from 26 April. The solid lines indicate the maximum likelihood estimates of relative frequencies of the variants using collected data. The dashed lines represent the predicted variant frequencies after 13 June. Dotted lines indicate lower and upper bounds of 95% CI of estimated variant frequencies.

### Population average of relative instantaneous reproduction numbers

The population average of *R_RI_*, 1+*s*, with respect to strains labelled as others at calendar time *t* was estimated by 1+*s*_R.1_ *q*_R.1_(*t*_R.1_) +*s*_Alpha_ *q*_Alpha_(*t*_Alpha_)+*s*_Delta_ *q*_Delta_(*t*_Delta_). Since the maximum likelihood estimates of *s*_R.1_, *s*_Alpha_, and *s*_Delta_ can be affected by the log mean and log standard deviation of the lognormal serial interval distribution, sensitivity analyses of parameters *s*_R.1_, *s*_Alpha_, and *s*_Delta_ were performed by using the combinations of *μ* and *σ* sampled along the boundary of the 95% confidence area of the likelihood surface of the serial interval distribution [11,12]. As a result, we estimated the ranges of the maximum likelihood estimates of *s*_R.1_, *s*_Alpha_, and *s*_Delta_ to be 19–32%, 34–58%, and 70%–130%, respectively.

**Figure 2:**
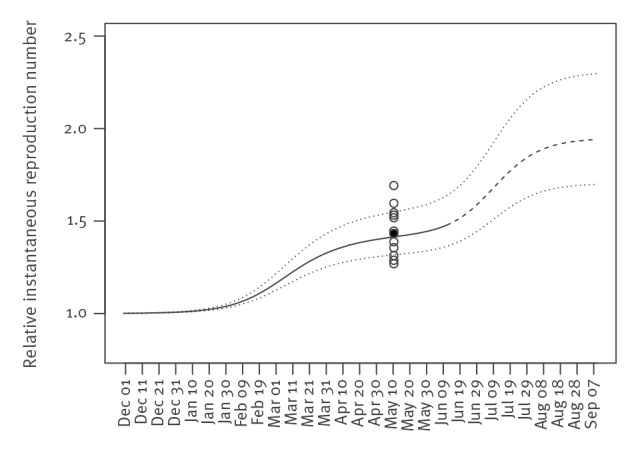
Temporal changes in the population average of the relative instantaneous reproduction numbers with respect to SARS-CoV-2 strains other than the R.1 variant or Alpha or Delta VOC, Japan, 1 December 2020 to 11 September 2021

SARS-CoV-2: severe acute respiratory syndrome coronavirus 2; VOC: variants of concern.

Figure 2 shows temporal changes in the population average of *R_RI_* with respect to strains other than the R.1 variant, and the Alpha or Delta VOC circulating in Japan. The population average of *R_RI_* stayed around one until the end of January 2021 since strains other than the R.1 variant, or Alpha or Delta VOC, were dominant around that time. From the beginning of February 2021, the population average of the *R_RI_* has been elevated due to the increase of frequencies of the Alpha VOC (Figure 1). The population average of the *R_RI_* values reached 1.20 (Serial interval sensitive range (SISR): 1.16–1.27) on 9 March 2021. The population average of relative transmissibility in May 2021 was consistent with the relative reproduction number of the Alpha VOC when compared with other variants circulating before Alpha, as estimated in Japan on 12 May 2021 [13], using the method proposed by Volz et al. [14]. From around the middle of June 2021, the population average of *R_RI_* became elevated because the Alpha VOC started to decrease in frequency and was replaced by the Delta VOC (Figure 1). The increase of the population average of the *R_RI_* will continue until the Alpha VOC is completely replaced by the Delta VOC by the middle of August 2021 (Figure 2).

The solid line (until 13 June 2021) and dashed line (from 14 June 2021) indicate the population average of *R_RI_* when *s*_R.1_ = 0.245, *s*_Alpha_ = 0.437, and *s*_Delta_ = 0.948, which are calculated by assuming the lognormal serial interval distribution with *μ* = 1.38 and *σ* = 0.563. The lower dotted line indicates the population average of the *R_RI_* when *s_R_*_.1_ = 0.190, *s*_Alpha_ = 0.335, and *s*_Delta_ = 0.703, which are the lower bounds of *s*_R.1_, *s*_Alpha_, and *s*_Delta_ calculated based of the 95% confidence area of the lognormal serial interval distribution. The upper dotted line indicates those when *s*_R.1_ = 0.319, *s*_Alpha_ = 0.580, and *s*_Delta_ = 1.30, which are upper bounds calculated in the same way. The filled circle represents the relative transmissibility of the Alpha VOC in Tokyo compared with other variants, mostly consisting of viruses circulating before the R.1 variant and Alpha VOC. Open circles represent those in Fukuoka, Miyagi, Okinawa, Hokkaido, Saitama, Chiba, Osaka, Kanagawa, Hyogo, Aichi, Kyoto prefectures from the top. It should be noted that the relative reproduction number compares the relative change in the instantaneous reproduction number compared with the baseline on 1 December 2020.

## Discussion and conclusions 

We have shown that the SARS-CoV-2 Delta VOC possesses greater transmissibility than the R.1 and the Alpha VOC. The *R_RI_* of the R.1 and the Alpha and Delta VOC with respect to other strains circulating in Japan were estimated at 1.25 (SISR: 1.16–1.27), 1.44 (SISR: 1.34–1.58), and 1.95 (SISR: 1.70–2.30), respectively. This means that the Delta VOC possesses almost 1.6 and 1.4 times higher transmissibility than the R.1 and the Alpha VOC, respectively. While the Alpha VOC has replaced other SARS-CoV-2 variants in Japan just over the last 5 months, it is very likely that it is just a matter of time for the Delta VOC to replace other variants, including Alpha.

Our results show that the replacement is likely to happen mostly before the start of the Tokyo Olympic Games on 23 July 2021. In terms of possible public health impact with respect to this event, the risk assessment should account for the fact that a substantial number of international visitors during the Games might be exposed to the Delta VOC, and increased mobility could help further spread COVID-19 caused by this variant with an elevated transmissibility around the world.

During the fourth wave in Japan, interventions had to be strengthened with the emergence of the Alpha VOC. Up until the third wave, the focused interventions on food service and drinking establishments without an explicit request to ‘stay home’ have been highly effective [15] prior to the introduction of the Alpha variant, even though vaccines were unavailable. However, because of elevated transmissibility with new variants, this strategy may not be effective to substantially reduce the reproduction number below the value of one. The two-dose vaccination coverage in Japan, which is 10.4% as at 6 July 2021 [16], should be increased rapidly.

## Supplementary Data

1161000 Supplementary Table 1

## Supplementary Data

30000 Supplementary Table 2

## Supplementary Data

20000 Supplementary Table 3

## References

[1] World Health Organization (WHO). SARS-CoV-2 Variants of Concern and Variants of Interest. Geneva: WHO; 2021. Available from: https://www.who.int/en/activities/tracking-SARS-CoV-2-variants/

[2] Rambaut A Holmes EC O’Toole Á Hill V McCrone JT Ruis C A dynamic nomenclature proposal for SARS-CoV-2 lineages to assist genomic epidemiology. Nat Microbiol. 2020;5(11):1403-7. 10.1038/s41564-020-0770-5 32669681 PMC7610519

[3] Tokyo Metropolitan Government. Updates on COVID-19 in Tokyo. Tokyo: Tokyo Metropolitan Government; 2021. Available from: https://stopcovid19.metro.tokyo.lg.jp/en

[4] Shu Y McCauley J. GISAID: Global initiative on sharing all influenza data - from vision to reality. Euro Surveill. 2017;22(13):30494. 10.2807/1560-7917.ES.2017.22.13.30494 28382917 PMC5388101

[5] Fraser C. Estimating individual and household reproduction numbers in an emerging epidemic. PLoS One. 2007;2(8):e758. 10.1371/journal.pone.0000758 17712406 PMC1950082

[6] Tokyo Metropolitan Government. Disaster Prevention Information. Tokyo: Tokyo Metropolitan Government; 2021. Available from: https://www.bousai.metro.tokyo.lg.jp/taisaku/saigai/1013388/1014026.html

[7] Pawitan Y. *In All Likelihood: Statistical Modelling and Inference Using Likelihood*. Croydon: Oxford University Press; 2013.

[8] Johnson SG. The NLopt nonlinear-optimization package. 2020. Available from: http://github.com/stevengj/nlopt

[9] Rowan T. *Functional Stability Analysis of Numerical Algorithms.* Ph.D. Thesis. Austin: University of Texas, Austin; 1990.

[10] Campbell F Archer B Laurenson-Schafer H Jinnai Y Konings F Batra N Increased transmissibility and global spread of SARS-CoV-2 variants of concern as at June 2021. Euro Surveill. 2021;26(24):2100509. 10.2807/1560-7917.ES.2021.26.24.2100509 34142653 PMC8212592

[11] Nishiura H Linton NM Akhmetzhanov AR. Serial interval of novel coronavirus (COVID-19) infections. Int J Infect Dis. 2020;93:284-6. 10.1016/j.ijid.2020.02.060 32145466 PMC7128842

[12] Piantham, C., Linton, N. M., Nishiura, H., & Ito, K. Estimating the elevated transmissibility of the B.1.1.7 strain over previously circulating strains in England using GISAID sequence frequencies. *medRxiv*. 2021. doi:10.1101/2021.03.17.21253775

[13] Ministry of Health. Labour and Welfare. Reports from Advisory Board Members for COVID-19. Tokyo: Ministry of Health, Labour and Welfare; 2021. Available from: https://www.mhlw.go.jp/stf/seisakunitsuite/bunya/0000121431_00256.html

[14] Volz, E., Mishra, S., Chand, M., Barrett, J. C., Johnson, R., Geidelberg, L., et al. Transmission of SARS-CoV-2 Lineage B.1.1.7 in England: Insights from linking epidemiological and genetic data. *medRxiv*, 2020.2012.2030.20249034. doi:10.1101/2020.12.30.20249034

[15] Nakajo K Nishiura H. Assessing Interventions against Coronavirus Disease 2019 (COVID-19) in Osaka, Japan: A Modeling Study. J Clin Med. 2021;10(6):1256. 10.3390/jcm10061256 33803634 PMC8003080

[16] Government CIOs' portal, Japan. COVID-19 Vaccination in Japan. 2021. Available from: https://cio.go.jp/en/index.php

